# *Actinidia deliciosa* Mitigates Oxidative Stress and Changes in Pancreatic *α*-, *β*-, and δ-Cells and Immunohistochemical and Histological Architecture in Diabetic Rats

**DOI:** 10.1155/2022/5224207

**Published:** 2022-04-27

**Authors:** Fatma M. El-Demerdash, Yousra Talaat, Nora F. Ghanem, Wenyi Kang

**Affiliations:** ^1^Department of Environmental Studies, Institute of Graduate Studies and Research, Alexandria University, Alexandria, Egypt; ^2^Department of Zoology, Faculty of Science, Kafrelsheikh University, Kafr El-Sheikh, Egypt; ^3^National R & D Center for Edible Fungus Processing Technology, Henan University, Kaifeng 475004, China

## Abstract

The present study evaluated the antioxidant capacity and antidiabetic effect of *Actinidia deliciosa* in diabetic rats. Rats were grouped as follows: control, *Actinidia deliciosa* aqueous extract (ADAE, 1 g/kg, daily and orally), streptozotocin (STZ, 50 mg/kg BW, single intraperitoneal dose), and STZ plus ADAE, respectively. Twenty-eight components were detected by GC-MS analysis with high phenolic contents and high DPPH scavenging activity. In vivo results revealed that rats treated with STZ showed a highly significant elevation in blood glucose and a decrease in insulin hormone levels. Thiobarbituric acid-reactive substances and hydrogen peroxide levels were elevated, while bodyweight, enzymatic, and nonenzymatic antioxidants were significantly decreased. Furthermore, histopathological and immunohistochemical insulin expression, besides ultrastructure microscopic variations (*β*-cells, *α*-cells, and δ-cells), were seen in pancreas sections supporting the obtained biochemical changes. Otherwise, rats supplemented with ADAE alone showed an improved antioxidant status and declined lipid peroxidation. Moreover, diabetic rats augmented with ADAE showed significant modulation in oxidative stress markers and different pancreatic tissue investigations compared to diabetic ones. Conclusively, ADAE has a potent antioxidant and hypoglycemic influence that may be utilized as a health-promoting complementary therapy in diabetes mellitus.

## 1. Introduction 

Diabetes mellitus (DM) is a group of metabolic disorders, which is described by an absolute or relative shortage in insulin secretion and/or insulin action accompanied by persistent hyperglycemia and perturbations of carbohydrate, lipid, and protein metabolism [1]. DM manifests all over the world but is widespread in developing countries with low and middle income where approximately 80% of diabetic deaths take place. Streptozotocin (STZ) is a wide-spectrum antibiotic that induces specifically *β*-cell necrosis in pancreatic islets [2]. Generally, it is presumed that STZ is taken up through the “cell membrane GLUT2 glucose transporter” and leads to DNA and protein alkylation and consequent *β*-cell death, and it acts as a nitric oxide giver that may relate to its cytotoxicity [3].

Traditional remedies derived from medicinal plants displayed a vital and important role in the control of DM and its complications [4]. Owing to their active ingredients, medicinal herbs have been reported to acquire some characteristic properties like pancreatic *β*-cell regeneration, insulin-releasing, and combating the problem of insulin resistance. One of these plants is *Actinidia deliciosa* (AD) or kiwifruit that belongs to the Actinidiaceae family and is home-grown in China. AD is reputed as an essential source of polyphenols, dietary fibers, protein, calcium, iron, and various vitamins, especially vitamin C [5, 6]. AD has many biological activities, including antioxidant, anticancer, amendment of platelet accumulation, regulation of lipids level, lowering blood pressure, and modification of inflammation process [5, 7, 8]. Therefore, the present work is designed to investigate the antihyperglycemic and antioxidant potentiality of *Actinidia deliciosa* aqueous extract against oxidative stress and pancreatic deterioration in diabetic rats.

## 2. Materials and Methods

### 2.1. Materials

*Actinidia deliciosa* (kiwifruit) was purchased from the local market in Alexandria, Egypt, and identified by the Botany Department, Faculty of Science, Alexandria University. Streptozotocin was procured from Sigma Chemical Company, USA.

### 2.2. Preparation of Lyophilized *Actinidia deliciosa* Aqueous Extracts

Kiwifruits were prepared based on the method of Bursal and Gülçin [9]. 100 grams was ground in a mill into fine particles, mixed with 250 distilled water, stirred at 25°C for one day, and filtered on filter paper. The obtained filtrates were frozen and then lyophilized, and the lyophilized powder was kept at −80°C until used.

### 2.3. GC-MS Analysis of *Actinidia deliciosa*

Chemical constituents of lyophilized *Actinidia deliciosa aqueous extract* were identified using a Thermo Scientific GC/MS version (5) 2009 system with TG-5MS column (30 m × 0.32 mmID). The components of the ADAE were identified by mass fragmentation patterns and compared with the National Institute of Standards and Technology (NIST) mass spectral database (version 2), and their relative percentages were measured based on GC peak areas.

### 2.4. Determination of DPPH Radical Scavenging Activity and Total Phenolic Content of *Actinidia deliciosa*

The DPPH-free radical scavenging activity and total phenolic contents of ADAE were evaluated by the previously described methods [10, 11].

### 2.5. Diabetes Induction

Rats were fasted throughout the night and provided with water freely. Diabetes was induced in male rats using a single injected intraperitoneal dose of 50 mg/kg body weight of streptozotocin freshly dissolved in 0.01 M citrate buffer (pH 4.5) [12]. Rats were permitted to drink glucose solution (5% w/v) overnight to avert hypoglycemia which might be caused by streptozotocin, and then the animals were fed with a normal diet during the study. A week later, a characteristic sign of diabetes was confirmed by the existence of hyperglycemia in which the blood glucose level was higher than 250 mg/dL [13].

### 2.6. Animals and Experimental Protocol

Twenty-four male albino Wistar rats weighing 160–170 g were procured from the animal house of the Institute of Graduate Studies and Research, Alexandria University. Animals were handled following the principles of laboratory animal care as contained in the National Institutes of Health (NIH) guide for laboratory animal welfare, and the experimental protocol was authorized by the Local Ethics Committee and Animals Research (AU14-190323-2-7). The male rats were hosted in stainless steel bottomed wire cages and kept at a temperature of 22 ± 2°C and relative humidity of 40–60% with a 12 h/12 h light/dark cycle and free access to pellet regimen and water ad libitum. After fourteen days of acclimatization, rats were randomly allocated to four groups with six animals each: control, *Actinidia deliciosa* aqueous extract (ADAE, 1 g/kg/day orally for 30 days) [14], streptozotocin (50 mg/kg, i.p., single dose) [12], and STZ (diabetic rats) plus ADAE, respectively. By the end of the experimental period, the rats were anesthetized using isoflurane and then euthanized, and blood and pancreas were taken for furthermore analysis.

### 2.7. Blood and Tissue Samples

Blood samples were individually collected from the aorta of each rat in nonheparinized glass tubes. Blood samples were allowed to stand for 15 minutes at room temperature to clot before being centrifuged at 3000 × g for 15 min. Serum samples were kept at −80°C until being used. Pancreas tissues were immediately removed, weighed, and rinsed by 0.9% cold saline solution, homogenized (10% w/v) in ice-cold 0.01 M sodium phosphate buffer (pH 7.4) containing 1.15% KCl using a chilled glass Teflon Potter-Elvehjem Tissue Grinder tube, and then centrifuged at 10,000 × g for 20 minutes at 4°C. The resultant supernatants were utilized for different assays determinations.

### 2.8. Determination of Oxidative Stress Markers

Thiobarbituric acid-reactive substances (TBARS), hydrogen peroxide (H_2_O_2_), and reduced glutathione (GSH) were measured in the pancreatic homogenate. Also, the activities of superoxide dismutase (SOD, EC 1.15.1.1), catalase (CAT, EC 1.11.1.6), glutathione peroxidase (GPx, EC 1.11.1.9), glutathione reductase (GR, EC 1.6.4.2), and glutathione S-transferase (GST, EC 2.5.1.18) were assessed using kits from Biodiagnostic, Egypt.

### 2.9. Determination of Body Weight, Glucose, C-Peptide, and Insulin Hormone Levels

Serum insulin level was analyzed using an ultrasensitive rat insulin ELISA kit (Mercodia AB, Uppsala, Sweden) and a multiplate ELISA reader (Biorad-680, BIORAD Ltd., Japan), while glucose level was determined using kits (Biodiagnostic, Egypt). Serum was also used to investigate C-peptide levels using commercial rat ELISA kits (ALPCO, Catalog No. 80-CPTRT-E01).

### 2.10. Histopathological Examinations

Pancreas tissues were put in formalin solution, and sequent paraffin sections were stained using hematoxylin and eosin stain to investigate the histological alterations. Then, slides were photographed utilizing a light microscope (Olympus BX 41, Japan).

### 2.11. Immunohistochemistry Examination

The immunohistochemistry stain method used for staining the pancreas tissues was performed according to Cuello et al. [15]. Ultrasemithin section from Araldite capsule was mounted on coated slides and stained for insulin detection in insulin-producing cells. The slides for each animal group were rinsed in acetone and treated with serial of descended alcohol concentrations. The slides were treated with hydrogen peroxide, incubated in a peroxide protein blocker, and then incubated with primary antibody (monoclonal insulin antibody) obtained from Cell Signaling^@^ (USA) at 4°C overnight using a humid chamber. On the 2nd day, incubation of the slides with peroxidase-conjugated secondary antibodies for one hour at room temperature took place followed by incubation with media containing Avidin-Biotin complex for 30 minutes. The slides were washed and stained with DAB (3, 3 diaminobenzidine tetrahydrochloride) as chromogen using kits obtained from R&D Systems, Inc. (USA). Then the slides were washed, dehydrated, mounted, and covered by coverslips for demonstration under the light microscopy.

### 2.12. Transmission Electron Microscopy Examination (TEM)

Pancreas specimens were rapidly cut into small pieces about (1 mm^3^), then fixed in 2.5% glutaraldehyde, buffered in 0.1 M sodium cacodylate at 4°C, postfixed for one day in osmium tetroxide, then dried up in ascending grades of ethyl alcohol, treated with propylene oxide and embedded in EPON, and sectioned by ultramicrotome. Thick sections (1 *µ*m thick) were fitted on glass slides and stained with toluidine blue to be examined with a light microscope. Ultrathin sections (60–70 nm thick) rode up on copper grids and were stained with uranyl acetate and lead citrate [16]. Sections were examined and photographed using Joel JEM-1010 transmission electron microscope (TEM) at Electron Microscope Unit, Faculty of Science, Alexandria University.

### 2.13. Statistical Analysis

Data from various groups were represented as means ± standard errors (SEM) and then analyzed utilizing SPSS software (version 22, IBM Co., Armonk, NY). Comparison between groups was made through one-way ANOVA followed by Tukey's post hoc test. *P* value ≤0.05 was approved to be significant.

## 3. Results

### 3.1. GC-MS

The GC-MS analysis of ADAE (Table 1) displayed the existence of various bioactive components that could contribute to the antioxidant and therapeutic benefits of kiwifruit. The recognition of the phytochemical components was verified based on the peak area, retention time (RT), and molecular formula.

### 3.2. In Vitro Antioxidant Capacity

In this study, ADAE effectively inhibited DPPH-free radical scavenging activity with an IC_50_ value of 2.3 versus Vit C with an IC_50_ value of 3.5 mg/ml. The DPPH radical scavenging percentages of different concentrations (0.25–10 mg/mL) of ADAE extract were 30.1, 37.7, 49.7, 59.2, 63.8, 76.3, and 88.4%, respectively. Moreover, the total phenolic content was found to be equal to 12.8 ± 0.08 *µ*g GAE/mg DW.

### 3.3. Body Weight, Glucose, and Insulin Level

No signs of morbidity or mortality were observed in STZ-treated rats during the study period. Blood glucose concentration was markedly increased, while C-peptide, insulin hormone level, and body weight, as well as body weight gain, were significantly decreased as compared to control. However, *Actinidia deliciosa* aqueous extract supplementation in diabetic rats alleviates the changes detected in these parameters comparable to the diabetic group. *Actinidia deliciosa* alone did not cause any significant change in the measured parameters (Table 2).

### 3.4. Oxidative Stress Markers

Results revealed a significant (*P* < 0.05) increase in TBARS and H_2_O_2_ levels and a decrease in GSH content and SOD, CAT, GPx, GR, and GST activities in diabetic rats' pancreatic homogenate versus control, while diabetic rats treated with ADAE displayed a significant amelioration as related to diabetic ones. Supplementation with ADAE alone reduced the concentrations of TBARS and H_2_O_2_ and induced GSH, SOD, CAT, GPx, GR, and GST in the pancreas homogenate (Table 3).

### 3.5. Pancreas Histopathology and Immunohistochemistry

**Figure 1:**
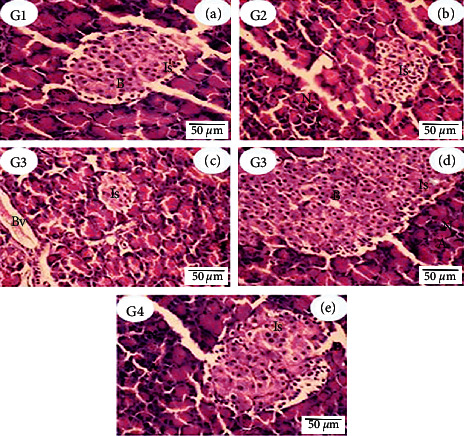
Histopathological examination photomicrograph of the pancreatic section of control rats (G1, (a)) and *Actinidia deliciosa* (G2, (b)) groups showed the normal structure of islet of Langerhans cells (Is) gland, clumping of *β*-cells surrounded by small nuclei cells situated at the periphery of the gland, and acini gland (A) having epithelial cells with dark chromatic nuclei (N). Rats treated with STZ (diabetic rats) (G3, (c)) showed atrophy of islet (Is) Langerhans with reduction of the *β*-cells. The acinar cells stained strongly are arranged in lobules with prominent dark chromatin nuclei with marked dilation of blood vessels (Bv). (G3, (d)) Diabetic rat pancreas showed high proliferating *β*-cells (B), which crowded in the islet (appeared as small glands in structure). The large islet (Is) is surrounded by acini gland (A) with normal architecture epithelial cells with dark chromatic nuclei (N). The pancreas of diabetic rats that received *Actinidia deliciosa* (G4, (e)) showed normal proportions of the islet of Langerhans cells (Is) with mild edema (E) (H&E stain, bar = 50 *µ*m).

Light microscopic investigation of the pancreas section of rats from control (G1) and ADAE (G2) showed normal histological architecture of the endocrine portion islets of Langerhans with clumping of *β*-cells surrounded by small nuclei cells situated at the periphery and the exocrine portion represented by acini gland having epithelial cells with dark chromatic nuclei. In contrast, STZ-induced diabetes (G3) in rats showed atrophy of Langerhans islets with reduction of *β*-cells, where the acinar cells of exocrine portions were stained strongly and arranged in lobules with prominent dark chromatin nuclei with a marked expansion of blood vessels and normal interlobular duct. Also, high proliferating *β*-cells in some cases, which crowded in the islets, appeared as small glands in the structure and large islets surrounded by acini gland with normal architecture epithelial cells with dark chromatin nuclei. Moreover, the diabetic rats which received ADAE (G4) showed that islets of Langerhans cells of the pancreas are present in their normal proportion with mild edema, which appeared as regenerative *β*-cells and acinar cells of the exocrine portions of the pancreas, stained strongly and arranged in lobules with prominent dark chromatin nuclei (Figure 1).

**Figure 2:**
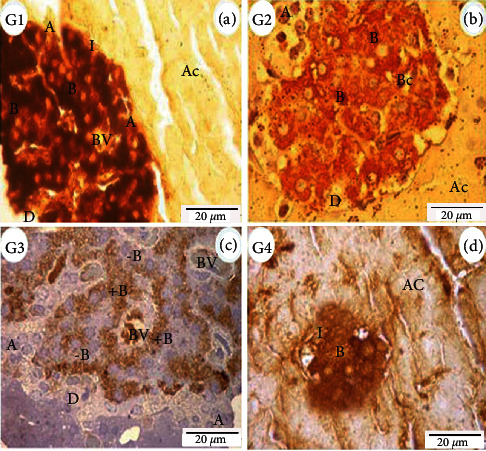
Immunohistochemical photomicrograph showed normal rat pancreas (G1, (a)) with dark intensity positive immunostaining of insulin antibody in islet (I) and presence of *β*-cells (B) and negative in Alpha- (A) and Delta-cells (D) as well as acini (AC) and blood vessels (BV). The pancreas of rats administered with *Actinidia deliciosa* (G2, (b)) shows the moderate positive immunostaining of insulin in most *β*-cell expressed in the cytoplasm and absent insulin in the area of Alpha- (A) and Delta-cells (D) and presence of blood capillaries (BC) and negative epithelial cells of pancreatic acini (AC). Diabetic rats (G3, (c)) show proliferating and crowded *β*-cells in islet with a decrease of the positive immunostaining of the insulin at periphery cells (+B), negative blue *β*-cell (−B), D-cell, and *α*-cell (A), and increased dilated blood vessels (BV). Diabetic rats supplemented with *Actinidia deliciosa* (G4, (d)) show improved *β*-cell with moderate positivity diffuse immunostaining of the insulin on *β*-cells (+B).

The positive reaction of insulin receptor appeared as brown granules scattered in the cytoplasm of *β*-cell in the pancreatic islet and absent in nuclei of *β*-cell. An intense positive insulin expression was observed in the control (G1) and ADAE (G2) groups. The moderate reaction of insulin expression in the most crowded *β*-cell and area of the blue absent reaction of the pancreatic islet was seen in the diabetic group (G3). A strong reaction of insulin expression in all *β*-cells appeared in the diabetic rats' group supplemented with ADAE (G4) (Figure 2).

### 3.6. Transmission Electron Microscopic Investigation

**Figure 3:**
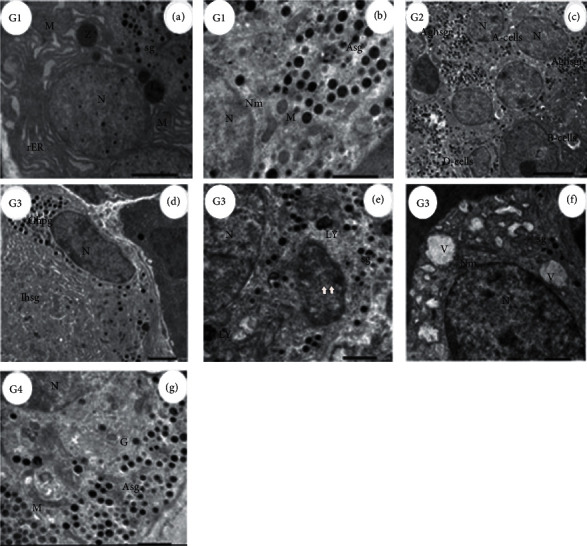
Electron micrograph of Alpha-cells (*α*-cells) in different groups. (G1, (a)) Electron micrograph of rat pancreas showing a normal portion of exocrine with obvious nuclei (N) surrounded by rER containing zymogen granules (Z), the section showing also part of the endocrine (islets of Langerhans) revealing *α*-cells secretory granules (sg) and mitochondria (M). Scale bar = 2.0 *µ*m, ×4000. (G1, (b)) Higher power view of control (G1) *α*-cells illustrating part of nuclei (N) with double nuclear membrane (Nm) and mitochondria (M) plus glucagon hormone-secreting granules (Asg). Scale bar = 1.0 *µ*m, ×8000. (G2, (c)) ADAE-administered rats islets of Langerhans show two A-cells with nucleus (N) and normal glucagon hormone secretory granules (Aghsgr). The section showing also B-cells and D-cell (scale bar = 5.0 *µ*m, ×1500). (G3, (d)) Diabetic rats *α*-cells with enlarged nuclei (N) and glucagon hormone-producing (Ghpg) + very few *β*-cells of insulin hormone-secreting granules (Ihsg) without a nucleus (scale bar = 2.0 *µ*m, ×2500). (G3, (e)) High power view of *α*-cells in the pancreas of rats treated with STZ (G3) islets of Langerhans revealing part of the nucleus (N), cytoplasm with secretory granules (sg), autophagy (arrows), and lysosomes (LY) (scale bar = 1.0 *µ*m, ×6000). (G3, (f)) Illustration of damaged *α*-cells, part of the nucleus (N), with double nuclear membrane (NM), absence of organoids in the cytoplasm, numerous vacuoles (V), and few secretory granules (sg) compared with control (scale bar = 1.0 *µ*m, ×5000). (G4, (g)) Diabetic rat pancreas treated with ADAE revealing normal *α*-cells part of the nucleus (N) + cytoplasmic secretory granules (Asg), Golgi apparatus (G), and mitochondria (M) (scale bar = 1.0 *µ*m, ×6000).

Electron micrographs of the pancreas of the control group (G1) showed normal exocrine portion pancreatic acini, a section of *α*-cell illustrating the glucagon hormone responsible for elevating the glucose level in the plasma. The pancreas of rats treated with ADAE (G2) revealed a normal nucleus of *α*-cell, cytoplasmic secretory granules, Golgi apparatus, and mitochondria. A-cell of diabetic (G3) rats showed some changes in the nuclei, glucagon hormone-producing granules, cytoplasmic secretory granules, mitochondria, and lysosomes, while in diabetic rats treated with kiwi (G4), part of the nucleus with the double nuclear membrane and marked dilated and fragmented rER appeared (Figure 3).

**Figure 4:**
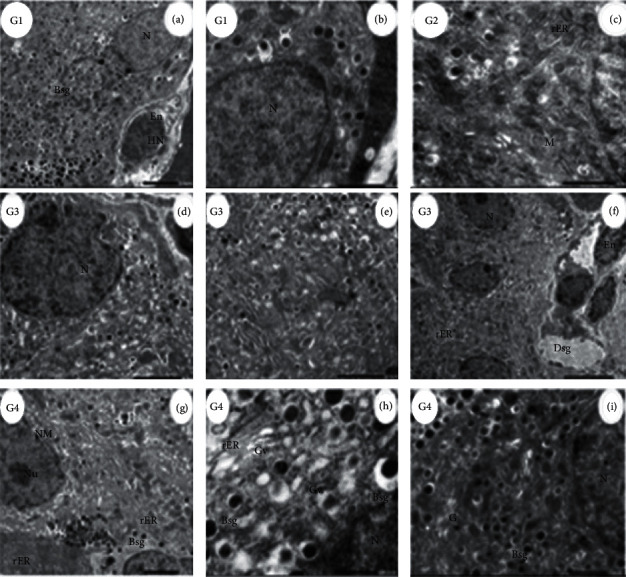
Electron micrograph of Beta-cells (*β*-cells) in different groups. (G1, (a)) Control rat pancreas, islets of Langerhans denoting normal *β*-cells with nucleus (N), and normal secretory granules (Bsg) characterized by the electron-dense core and surrounded by a clear zone (cz) and area of A-cells secretory granules (Asg) and blood capillary (Bc) lined by large endothelial cell (En) with heterochromatic nuclei (HN) (scale bar = 2.0 *µ*m, ×3000). (G1, (b)) Normal *β*-cells depicting the nucleus (N) (scale bar = 1.0 *µ*m, ×8000). (G2, (c)) *β*-cells in islets of Langerhans of rat pancreas treated with ADAE showing part of the nucleus (N) with double nuclear membrane (NM), *β*-cells secretory granules (sg), Golgi apparatus (G) rER, and mitochondria (M) (scale bar = 1.0 *µ*m. ×8000). (G3, (d)) Diabetic rat pancreas, *β*-cells with abnormal nuclei (N) with insulin hormone-secreting granules (Ihsg), mitochondria (M), and Golgi apparatus (G) (scale bar = 2.0 *µ*m. ×2000). (G3, (e)) *β*-cells with pyknotic nuclei (PY) pyknosis + granules without secretion (gws) (scale bar = 2.0 *µ*m. ×4000). (G3, (f)) *β*-cells with nuclei (N) with few granules (G) small in size and vacuolated rER + blood capillaries lined by 3 endothelial cells (En) (scale bar = 5.0 *µ*m, ×1500). (G4, (g)) Islets of Langerhans of rat pancreas treated with STZ + ADAE denoting *β*-cells with segregated nuclear membrane (NM) and obvious nucleolus (Nu) and insulin-secreting granules with migrating granule apart of the cell (Bsg), rER, and mitochondria (M), glucagon hormone-secreting granules (Asg) without a nucleus, and *ẟ*-cells with somatostatin hormone-secreting granules (Dsg) apart of exocrine and rER (scale bar = 2.0 *µ*m, ×3000. (G4, (h) and (i)) *β*-cells of diabetic rat + ADAE showing part of the nucleus (N), Golgi vesicles (Gv), rER, and insulin hormone-secreting granules (Bsg) in the cytoplasm) (scale bar = 500.0 nm, ×15000; scale bar = 1.0 *µ*m, ×6000).

Islets of Langerhans of control (G1) rat (the endocrine portion) denote normal *β*-cells (B) with normal nucleus, secretory granules producing insulin hormone. In higher magnification, the micrograph depicting the changes in the *β*-cell nucleus and secretory granules secretes insulin hormone to lower the glucose level in the plasma. *β*-cell in the pancreas of rats treated with ADAE (G2) shows normal nucleus and cytoplasmic secretory granules, Golgi apparatus, and mitochondria, while in diabetic (G3) rats, in islets of Langerhans, there was *β*-cell with swollen nuclei with nuclear pockets and peripheral nucleoli with insulin hormone-secreting granules, mitochondria, and Golgi apparatus. Also, *β*-cell appeared with pyknotic nuclei, granules without secretion of insulin. Another figure showed *β*-cells with few insulin-producing granules, vacuolated rER, and blood capillary. In islets of Langerhans of diabetic rats supplemented with ADAE (G4) showing improvement of *β*-cell, an enlarged portion of *β*-cell shows a small number of rER without granules (Figure 4).

**Figure 5:**
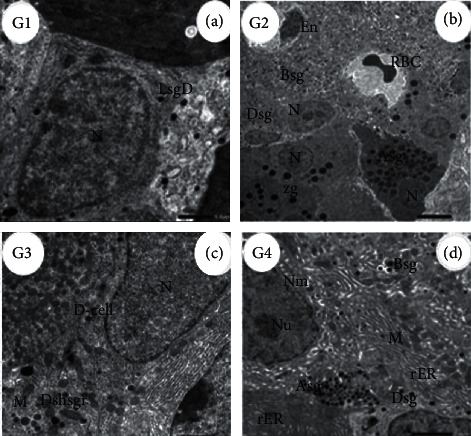
Electron micrograph of Delta-cells (*ẟ*-cells) in different groups. (G1, (a)) Control rat pancreas in islets of Langerhans showing *ẟ*-cells with large oval nuclei (N) and fewer secreting granules (LsgD), which secrete somatostatin hormones (scale bar = 1.0 *µ*m, ×5000). (G2, (b)) Rats treated with ADAE, pancreas denoting *ẟ*-cells with less cytoplasmic secretory granules (DSg), *α*-cells with nucleus (N) and glucagon hormone-secreting cytoplasmic granules (Asg), *β*-cells with nucleus (N) and less insulin hormone-secreting granules (Bsg) and blood capillary lined with endothelial cells (En), and other without lining endothelial cell containing RBCs besides the islets surrounded by exocrine cells with zymogen granules (zg) and nuclei (N) (scale bar = 5.0 *µ*m, ×1000). (G3, (c)) Diabetic rats showed *ẟ*-cells with few somatostatin hormone-secreting granules (Dshsgr), which inhibit the secretion of insulin and glucagon hormones, euchromatic nuclei, and mitochondria (M) (scale bar = 1.0 *µ*m, ×5000). (G4, (d)) Islets of Langerhans of rat pancreas treated with STZ + ADAE denoting *β*-cells with segregated nuclear membrane (NM) and obvious nucleolus (Nu) and insulin-secreting granules with migrating granule apart of the cell (Bsg), rER, and mitochondria (M), glucagon hormone-secreting granules (Asg) without a nucleus, and *ẟ*-cells with somatostatin hormone-secreting granules (Dsg) apart of exocrine and rER (scale bar = 2.0 *µ*m, ×3000).

Islets of Langerhans in control (G1) rat pancreas illustrate the δ-cell with large nuclei and fewer secreting granules. The pancreas of rats received ADAE (G2) denoting δ-cell with less cytoplasmic secretory granules and nucleus. Diabetic group rats (G3) revealing δ-cell with few somatostatin hormone-secreting granules, which inhibit the secretion of insulin and glucagon hormones. In the pancreas of diabetic rats supplemented with ADAE (G4), there are glucagon secreting granules without nucleus and δ-cell with few granules for somatostatin hormone production (Figure 5).

## 4. Discussion

Few studies have indicated the efficacy of *Actinidia deliciosa* as complementary medicine in DM. So, the current study was extended to determine the antioxidant and hypoglycemic efficacy of ADAE in STZ-induced diabetic rats. Insulin is a well-known hormone that regulates plasma glucose level homeostasis and promotes glycogenesis and glucose consumption [17]. The observed alterations in glucose and insulin hormone in STZ-treated rats are related to the destruction of pancreatic *β*-cells accompanied by hyperglycemia, hyperlipidemia, and weight loss [18]. Also, the reduction in insulin level that was observed in diabetic rats finally results in the deterioration in glucokinase activity which is an insulin-sensitive enzyme [17]. Further, glucokinase deficiency in diabetic rats reduced glycolysis and declined exhaustion of glucose for energy production [19]. In addition, Jones and Hattersley and Khaled et al. confirmed that C-peptide was formed in pancreatic *β*-cells by cleavage of proinsulin to equal amounts of C-peptide and insulin, and they declared that C-peptide can be used to assess endogenous insulin secretion [20, 21].

Damage in the pancreatic *β*-cell is clinically linked to the development of diabetes [22]. In this circumstance, STZ-induced hyperglycemia leads to the formation of reactive oxygen species (ROS), which can deplete antioxidant status and induce oxidative stress that can lead to the development of diabetes mellitus complications, and this is in harmony with the present study [4, 23]. Many factors can cause oxidative stress in diabetes, including glucose autoxidation leading to the production of free radicals. Other parameters include cellular oxidation/reduction imbalance and depression in enzymatic and nonenzymatic antioxidants, which are so important in the defense against free radicals generated in diabetes and are used as indicators of oxidative stress. The significant decrease in enzymatic and nonenzymatic antioxidants in diabetic rats might be because of the noted excess generation of ROS by STZ [17, 23, 24]. Glutathione plays a crucial key role in cellular defense versus xenobiotic toxicity because of its thiol group besides its action as a substrate for GPx and GST antioxidant enzymes [25]. The diminution of GSH content in diabetic rats might be attributed to its consumption in protecting versus oxidative stress in diabetes [22]. Moreover, GSH synthesis might be affected through the inhibition of glutathione-synthase and glucose 6-phosphate dehydrogenase activities. SOD catalyzes superoxide anion to O_2_ and H_2_O_2_, which is then reduced to water by the CAT enzyme. The decrease in both SOD and CAT activities in STZ-treated rats might be because of the excess generation of ROS by STZ [3]. GPx preserves the cellular membrane lipids from oxidative injury and catalyzes the reaction of hydroperoxides with GSH to form oxidized glutathione (GSSG). Also, the alteration in GR activity in diabetic rats was to recompense the decreased GSH contents via reducing GSSG that might be increased because of high free radicals' concentrations, while GST critically contributes to the elimination of toxic components and converting them to nontoxic metabolites [26, 27]. So, antioxidant enzymes, which prohibit free radicals chain reaction, are so important in restoring the glucose level in diabetic rats.

In the current study, histopathological evaluation of pancreatic tissue of diabetic rats showed atrophy of islets of Langerhans, and this is in line with the previous findings [28, 29] that reported progressive destruction of *β*-cells and loss of their histological architecture as well as extensive destruction to islets of Langerhans and decreased dimension of islets, respectively. Our results from immunohistochemical examination revealed that positive reaction of insulin receptor appeared as brown granules scattered in the cytoplasm of *β*-cells in the pancreatic islets and absent in nuclei of *β*-cells and other pancreatic cells appeared as a blue color as *α*, δ, and pancreatic acini. Intense positive insulin in normal moderate reaction in most crowded *β*-cells and absent reaction in the diabetic group as a blue color was observed [29]. Our results from transmission electron microscope study revealed that, in diabetic rat pancreas islets of Langerhans, the endocrine portion of the pancreas, there were *β*-cells with abnormal nuclei, swollen with nuclear pocket, other *β*-cells with pyknotic nuclei, granules without secretion of insulin, and very few *β*-cells of insulin hormone-producing granules. Similarly, the severity of diabetes in guinea pigs and the Chinese hamster is a function of reduced insulin synthesis and is directly related to the number of surviving functional *β*-cells [30, 31].

There has been a growing interest in replacing synthetic antidiabetic drugs with natural antioxidants from plant materials, especially due to the evidence of diabetes engaged with the enhanced free radicals' generation and decreased antioxidant potential [32]. Plants comprise a great variety of substances that possess antioxidant activity and can prevent the formation of advanced glycated end products (AGEs) and other diabetic complications associated with oxidative stress [33]. Since *Actinidia deliciosa* is known to possess various biological activities [34, 35], the GC-MS analysis of ADAE demonstrated the presence of various bioactive components that could contribute to its antioxidant and therapeutic benefits [36]. The antioxidant activities of phenolic compounds are due to their redox properties, and the phenol moiety assists them to act as “reducing agents, hydrogen donors, and singlet oxygen quenchers” [37].

Our results showed that ADAE administration reduced lipid peroxidation in diabetic rats, and this reflects its antioxidant capacity [34, 35]. It can defend against ROS toxicity either by preventing its formation or by ceasing its attack through sweeping the reactive metabolites [38]. The healthful effect of ADAE may be related to the interaction of its antioxidant components with xenobiotics besides the presence of vitamin C, which acts as a strong antioxidant [39]. Also, *Actinidia deliciosa* is an important precursor of flavonols, which protects cells from the harmful consequence of free radicals incriminated in STZ-treated rats [40]. Thus, *Actinidia deliciosa* could overcome pancreas damage via frustrating oxidative tissue injuries.

It is well-known that antioxidant enzymes are very important in the preservation of cell homeostasis against the harmful effect of ROS. The detected increment in antioxidant enzyme activities may be attributed to the decrease in ROS generation that is inhibited by ADAE. Also, escalation in GSH content helps in ROS removal, preservation of cell integrity, and cellular components versus oxidation via glutathione redox cycle due to its reducing features and its prominent role in cellular metabolism. Furthermore, kiwifruit is an important dietary source of natural antioxidants as well as natural inhibitors of pancreatic lipase and *α*-glucosidase [34]. Similarly, administration of hesperetin, a citrus flavonoid, attenuates hyperglycemia and dyslipidemia through mitigating antioxidant competence in diabetic rats. Also, it improves regeneration of islet cells and further provokes insulin secretion by defending *β*-cells from free radicals' exploitation via prohibition of antioxidant enzymes glycation, indicating that flavonoids can alleviate oxidative injury and protect and repair pancreatic *β*-cells [17]. Moreover, flavonoids are extensively confirmed to create a valuable protective effect through activation of the antioxidant defense enzyme system [41]. Also, *Actinidia deliciosa* was found to be efficacious in attenuating the alterations in pancreatic tissue architecture and destruction of pancreatic *β*-cells in diabetic rats. Similarly, hesperetin and multiherbal mixture treatment protect the normal histological appearance of insulin-positive *β*-cells in diabetic rats [17, 42].

Consistent with the previous studies, the glucose level in diabetic rats administered with ADAE was decreased significantly indicating the antioxidant and ameliorating role of ADAE as it stimulates the potential release of insulin from *β*-cells of islets [43]. Also, flavonoids result in an enhancement in intracellular Ca^2+^ concentration and inhibition of ATP-sensitive K^+^ channels in pancreatic islets, a preliminary step in insulin synthesis [44]. The antidiabetic role of *Actinidia deliciosa* might be through the stimulation of the survival *β*-cells from islets Langerhans leading to additional insulin release. This was proved by the improved level of insulin in diabetic rats that received ADAE and recovers in the number of pancreatic *β*-cells as confirmed by our different histological, immunohistochemical, and ultrastructural examinations. So, this study is a successful attempt that introduces *Actinidia deliciosa* as a complementary therapy and superb antioxidant in diabetes disease.

## 5. Conclusion

In conclusion, *Actinidia deliciosa* exhibited antihyperglycemic and antioxidant potential, which acts by improving insulin secretion via pancreatic *β*-cell regeneration in diabetic rats. Furthermore, ADAE administration enhanced the antioxidant status by inhibiting ROS generation in the pancreas and protecting against the harmful effects of STZ. Moreover, it attenuates oxidative stress-induced variations in histopathological and immunohistochemical insulin expression, as well as ultrastructure microscopic investigations (*β*-cells, *α*-cells, and δ-cells) because of its potent antioxidant and antiradical activity. So, it might be used as a complementing therapy in DM.

## Figures and Tables

**Table 1:** Major components found in *Actinidia deliciosa* extract.

No.	Compound name	Molecular formula	Molecular weight
1	Ascorbic acid	C_38_H_68_O_8_	652
2	Methyl acetate	C_3_H_6_O_2_	74
3	Ethyl acetate	C_4_H_8_O_2_	88.11
4	Vitamin E	C_29_H_50_O_2_	430
5	Methyl propanoate	C_4_H_8_O_2_	88
6	Methyl 2-methylpropanoate	C_5_H_10_O_2_	102
7	Ethyl propanoate	C_5_H_10_O_2_	102
8	Ethyl 2-methylpropanoate	C_6_H_12_O_2_	116
9	Pentanal	C_5_H_10_O	86
10	Methyl butanoate	C_5_H_10_O_2_	102
11	*α*-Pinene	C_10_H_16_	136
12	Toluene	C_7_H_8_	92
13	Ethyl butanoate	C_6_H_12_O_2_	116
14	Hexanal	C_6_H_12_O	100
15	Methyl pentanoate	C_6_H_12_O_2_	116
16	(E)-2-Pentenal	C_5_H_8_O	98
17	p-Xylene	C_8_H_10_	106
18	m-Xylene	C_8_H_10_	106
19	(E)-3-Hexenala	C_6_H_12_	84
20	Oleic acid	C_6_H_12_O_6_	282
21	l-Penten-3-ol	C_5_H_10_O	86
22	l-Pentanol	C_5_H_12_O	88
23	p-Cymene	C_10_H_14_	134
24	2, 4-Hexadienal	C_6_H_8_O	96
25	ᾅ-Terpineol	C_10_H_18_O	154
26	E-9-Tetradecenoic acid	C_14_H_26_O_2_	226
27	Phytol	C_20_H_40_O	256
28	á-Sitosterol	C_29_H_50_O	414

**Table 2:** Body weights, blood glucose, and insulin levels in different groups.

Parameters	Control	ADAE	STZ	ADAE + STZ
Glucose (mg/dl)	108 ± 2.70^c^	101 ± 3.12^c^	356 ± 9.82^a^	195 ± 5.60^b^
Insulin hormone (*µ*U/ml)	11.43 ± 0.352^a^	10.93 ± 0.281^a^	6.52 ± 0.217^c^	9.38 ± 0.170^b^
C-peptide (ng/ml)	0.62 ± 0.002^a^	0.52 ± 0.01^a^	0.061 ± 0.003^c^	0.29 ± 0.002^b^
Initial body weight (g)	155 ± 2.69	159 ± 3.02	156 ± 2.61	155 ± 2.16
Final body weight (g)	217 ± 2.24^a^	224 ± 4.67^a^	174 ± 3.82^c^	198 ± 3.82^b^
Weight gain (g)	62 ± 4.21^a^	65 ± 5.96^a^	18 ± 3.97^c^	42 ± 2.78^b^

Values are expressed as means ± SE (*n* = 6) for each treatment group. ^a, b, c^Mean values within a row not sharing a common superscript letter were significantly different, *p* < 0.05. Statistically significant variations are compared as follows: the ADAE and STZ groups compared with the control group and the ADAE + STZ group compared with the STZ group. Weight gain = final body weight – initial body weight.

**Table 3:** Oxidative stress markers in the pancreatic homogenate of male rats in different groups.

Parameters	Control	ADAE	STZ	ADAE + STZ
TBARS (nmol/g tissue)	21.67 ± 0.78^c^	16.96 ± 0.63^d^	30.71 ± 1.08^a^	25.35 ± 0.78^b^
H_2_O_2_ (*µ*mol/g tissue)	30.41 ± 0.98^c^	24.36 ± 0.72^d^	42.84 ± 1.63^a^	35.07 ± 1.27^b^
GSH (mmol/mg protein)	15.54 ± 0.61^b^	18.32 ± 0.64^a^	8.43 ± 0.24^d^	11.34 ± 0.38^c^
SOD (U/mg protein)	4.14 ± 0.149^b^	5.02 ± 0.184^a^	2.28 ± 0.075^d^	3.38 ± 0.111^c^
CAT (*μ*mol/h/mg protein)	46 ± 1.65^b^	54 ± 1.03^a^	26 ± 0.86^d^	37 ± 1.08^c^
GST (*µ*mol/hr/mg protein)	0.77 ± 0.028^b^	0.93 ± 0.034^a^	0.41 ± 0.016^d^	0.61 ± 0.022^c^
GPx (U/mg protein)	0.785 ± 0.034^b^	0.931 ± 0.031^a^	0.440 ± 0.010^d^	0.610 ± 0.030^c^
GR (U/mg protein)	0.876 ± 0.034^b^	1.033 ± 0.035^a^	0.531 ± 0.019^d^	0.722 ± 0.028^c^

Values are expressed as means ± SE (*n* = 6) for each treatment group. ^a, b, c^Mean values within a row not sharing a common superscript letter were significantly different, *p* < 0.05. Statistically significant variations are compared as follows: the ADAE and STZ groups compared with the control group and the ADAE + STZ group compared with the STZ group.

## Data Availability

All the data used to support the findings of this study are included within the article.
